# PTMFusionNet: A Deep Learning Approach for Predicting Disease Related Post-translational Modification and Classifying Disease Subtypes

**DOI:** 10.1016/j.mcpro.2025.101009

**Published:** 2025-06-02

**Authors:** Jie Ni, Yifan Zhou, Bin Li, Xinting Zhang, Yuanyuan Deng, Jie Sun, Donghui Yan, Shengqi Jing, Shan Lu, Zhuoying Xie, Xin Zhang, Yun Liu

**Affiliations:** 1 Institute for Molecular Medical Technology, State Key Laboratory of Digital Medical Engineering, School of Biological Science and Medical Engineering, Southeast University, Nanjing, Jiangsu, China; 2 Department of Medical Informatics, School of Biomedical Engineering and Informatics, Nanjing Medical University, Nanjing, Jiangsu, China; 3 Institute of Biomedical Devices (Suzhou), Southeast University, Suzhou, Jiangsu, China; 4 Department of Information, The First Affiliated Hospital, Nanjing Medical University, Nanjing, Jiangsu, China; 5 Center for Data Management, The First Affiliated Hospital, Nanjing Medical University, Nanjing, Jiangsu, China; 6 Women and Children Department, The First Affiliated Hospital, Nanjing Medical University, Nanjing, Jiangsu, China

**Keywords:** post-translational modification, biomarker discovery, disease classification, graph convolutional network, feature weighting

## Abstract

With the advancement of technologies such as mass spectrometry, it has become possible to simultaneously perform large-scale detection of protein intensity and corresponding post-translational modification (PTM) information, thereby facilitating clinical diagnosis and treatment. However, existing PTM information is insufficient to fully integrate with protein expression data. We propose a deep learning method called PTMFusionNet, which predicts potential disease-related PTMs and integrates them with protein expression data to classify disease subtypes. PTMFusionNet includes two Graph Convolutional Network (GCN) models: the Layer-Attention Graph Convolutional Network (LAGCN) and the Feature Weighting Graph Convolutional Network (FWGCN). LAGCN is used to predict PTM potentiality scores, while FWGCN integrates these scores with protein expression data for disease subtype classification. Experimental results across three datasets (KIPAN, COADREAD, and THCA) demonstrate that PTMFusionNet outperforms benchmark algorithms in accuracy, F1 score, and AUC, highlighting its robustness in identifying critical PTM biomarkers and advancing disease subtyping.

## Highlights

• LAGCN learns prior knowledge from PTM-disease heterogeneous network.
• FWGCN uses feature weighting and prior knowledge for subtype classification.
• Integrating LAGCN and FWGCN improves classification accuracy and biomarker discovery.

## Graphical Abstract

**Figure:**
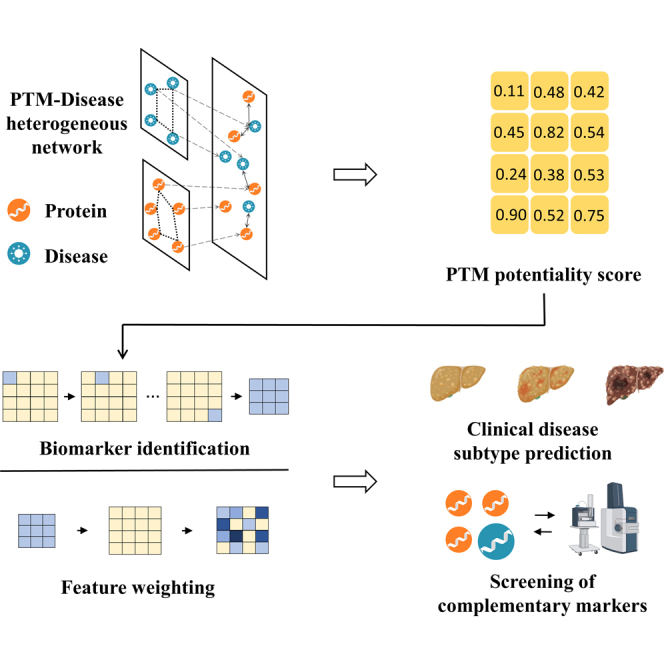

Post-translational modifications (PTMs) refer to the enzymatic addition of chemical groups to specific amino acid residues of a protein after translation, thereby altering the protein’s structure and function (1). PTMs, including phosphorylation, glycosylation, ubiquitination, methylation, and acetylation (2), play a crucial role in regulating protein activity, stability, localization, and interactions with other molecules (3, 4). As the final product of gene expression, the expression levels and modification states of proteins directly influence cellular functions and disease development (5).

Analyzing the proteome can reveal the molecular mechanisms of diseases, identify new biomarkers, and guide the development of personalized treatment plans (6, 7). Compared to the genome, the proteome has a higher correlation with pathological characteristics, making it more suitable for subtype classification based on pathological features (8). However, *in vitro* protein detection often falls short due to the limitations and high costs of detection methods.

With the advent of high-throughput detection technologies such as mass spectrometry, it is now possible to simultaneously perform large-scale detection of protein expression and PTMs (9). Artificial intelligence is increasingly playing a significant role in proteomics research. Protein expression data have the advantages of a high number of features and high throughput, but they also present challenges such as heterogeneity, noise, and information redundancy (10). PTMs have specific modification patterns that can more accurately reflect the specific states and progression of diseases (11, 12, 13). However, their dynamic and complex nature makes detection difficult, and different disease subtypes may have distinct PTM patterns (14). The limited coverage and accuracy of existing PTM databases further restrict their application in disease research (15).

In recent years, deep learning technologies have significantly advanced the development of precision disease diagnosis and treatment, particularly through groundbreaking methodological innovations in multimodal data fusion. Research teams have successively developed multiple innovative frameworks to optimize feature integration efficacy in biomedical data: Zhang *et al.* proposed an adaptive deep fusion network (ADFusion) based on deep equilibrium models, which demonstrated exceptional performance in cancer molecular subtyping through dual fusion mechanisms of joint features and complementary features (16); Meanwhile, Wang *et al.* proposed TMO-Net, an interpretable pretrained deep learning model integrating a cross-omics fusion network to learn latent variable associations across data modalities, enabling missing modality inference. Using multi-omics cancer datasets, TMO-Net applies to oncology tasks, including cancer subtype classification, metastasis prediction, drug response prediction, and prognosis forecasting (17).

However, existing studies predominantly focus on the synergistic analysis of multi-omics data such as mRNA, miRNA, and DNA methylation, while the clinical acquisition of such multimodal data often entails high technical costs and economic burdens. With the rapid advancement of high-resolution mass spectrometry, the clinical value of proteomics has become increasingly prominent (18, 19, 20). Nevertheless, current deep learning methodologies for proteomics data, particularly in fusing PTM information with feature expression optimization, have yet to form a systematic solution.

To address these issues, we have developed PTMFusionNet, which aims to integrate protein expression data with PTM markers for clinical analysis. PTMFusionNet performs various functions such as predicting potential PTM scores for diseases, utilizing these scores to guide disease subtype classification through protein expression data, selecting key protein markers, validating their accuracy through case studies, and verifying the accuracy of the top 20 PTMs ranked by potential scores through mass spectrometry analysis and literature review.

## Experimental Procedures

### Known Protein (PTM)-Disease Associations

In this study, we used two datasets: dataset1 and dataset2. Dataset1 contains broadly defined protein-disease known associations, sourced from the PhosphoSitePlus website (21). We downloaded the Disease-associated_sites.gz file from the website, which includes 1751 known protein-disease associations involving 1036 proteins and 391 diseases.

Dataset2 was downloaded from the PTMs related to human diseases (PTMD v1.0) database, an online resource providing information on PTMs and diseases (12). The original dataset contains 1950 known protein-disease associations, covering 749 proteins and 275 diseases. Additionally, the original dataset provides information on PTM types and PTM sites. We excluded all known protein-disease associations that did not involve any PTM sites, resulting in dataset2, which only includes disease association records involving PTMs. After filtering, we obtained 905 known associations involving 749 proteins and 275 diseases. In subsequent studies, depending on the specific objectives, one of the two datasets was selected as input to PTMFusionNet and denoted as A, with the detailed description as follows:

(1) $$A_{ij}=\left\{ \begin{array}{cc} 1 & \text{if}\;\text{protein}\;i\;\text{is}\;\text{associated}\;\text{with}\;\text{disease}\;j\\ 0 & \text{otherwise} \end{array}\right.$$

Due to the large number of known protein–disease associations and their extensive validation, dataset1 was primarily used in this study to determine hyperparameters and validate the effectiveness of PTMFusionNet. In subsequent processes for disease subtype classification and biomarker screening, PTM-disease known associations were used to explore whether disease progression is related to the occurrence of PTMs in one or more proteins.

### Protein Expression Datasets

The study utilized three protein expression datasets, obtained from The Cancer Genome Atlas (TCGA) via the Broad Institute's Genome Data Analysis Center (GDAC) Firehose (https://gdac.broadinstitute.org/), to validate the accuracy of the PTMFusionNet method in cancer classification. In the experiments presented in this study, the data underwent robust scaling pre-processing. Specifically, the COlorectal ADenocarcinoma and REctal ADenocarcinoma (COADREAD) dataset was bifurcated into COlorectal ADenocarcinoma (COAD) and REctal ADenocarcinoma (READ) categories. The Kidney Pan-Cancer (KIPAN) dataset was segregated into Kidney Chromophobe (KC), Kidney Renal Clear Cell Carcinoma (KRCC), and Kidney Renal Papillary Cell Carcinoma (KRPC) types. The Thyroid Carcinoma (THCA) dataset was classified into classical/common (papillary NOS) and other subtypes. Table 1 enumerates the patient count and label distribution for these datasets as well as the number of proteins concurrently present in the association data.

**Table 1:** Overview of datasets

Dataset	Categories	Number of proteins and shared proteins
COADREAD	COAD: 360, READ: 129	196, 28
KIPAN	KC: 63, KRCC: 475, KRPC: 215	178, 25
THCA	Classical: 160, Other: 62	175, 19

The table encapsulates data on protein expression. It specifically highlights proteins that are concurrently present in both protein expression datasets and PTM-disease association datasets.

### Similarity for Protein and Disease

In the process of analyzing the datasets and extracting potential association information, we calculated two different protein similarity measures: sequence similarity and Gaussian Interaction Profile kernel similarity. These calculations facilitate a comprehensive examination of the data, enabling the elucidation of inherent association patterns within protein profiles.

Protein sequence similarity, denoted as SSP, was calculated using the local sequence alignment algorithm from the pairwise2 module in Python. Specifically, we used the ‘pairwise2.align.localms’ function to compute pairwise similarity scores between sequences, with the parameters set as follows: match score: 2, mismatch penalty: −1, gap open penalty: −0.5, and gap extension penalty: −0.1. We recorded the highest score for each pair of sequences in the similarity matrix. For comparison purposes, we identified the maximum score in the matrix and normalized all scores to a range between 0 and 1.

Based on the hypothesis that functionally similar proteins are often associated with similar diseases, we calculated the similarity between proteins and diseases using the Gaussian interaction profile kernel function. Specifically, in the protein-disease association matrix A, we define the $i^{th}$ row $I\left(p_{i}\right)$ and the $j^{th}$ column $I\left(d_{j}\right)$ as interaction profiles for protein $p_{i}$ and disease $d_{j}$, respectively. We then calculated the Gaussian interaction profile kernel similarity between proteins (SGP) as

(2) $$SGP\left(p_{i},p_{j}\right)=\exp\left(-\;\rho_{p}{\left\|I\left(p_{i}\right)\;-\;I\left(p_{j}\right)\right\|}^{2}\right)$$

Similarly, the Gaussian interaction profile kernel similarity between diseases can be calculated as

(3) $$SGD\left(d_{i},d_{j}\right)=\exp\left(-\;\rho_{d}{\left\|I\left(d_{i}\right)\;-\;I\left(d_{j}\right)\right\|}^{2}\right)$$

where exp⁡(·) denotes the exponential function with the natural base, and the terms inside the parentheses represent the exponent. ${\left\|I\left(p_{i}\right)-I\left(p_{j}\right)\right\|}^{2}$ and ${\left\|I\left(d_{i}\right)-I\left(d_{j}\right)\right\|}^{2}$ represent the Euclidean norms of the vectors $I\left(p_{i}\right)-I\left(p_{j}\right)$ and $I\left(d_{i}\right)-I\left(d_{j}\right)$, respectively. $\rho_{p}$ and $\rho_{d}$ are the normalized kernel bandwidths for proteins and diseases, respectively, which can be calculated as

(4) $$\rho_{p}=\rho_{p}^{\prime }/\left(\left(1/np\right)\sum_{i=1}^{np}{\left\|I\left(p_{i}\right)\right\|}^{2}\right)$$

(5) $$\rho_{d}=\rho_{d}^{\prime }/\left(\left(1/nd\right)\sum_{i=1}^{nd}{\left\|I\left(d_{i}\right)\right\|}^{2}\right)$$

where np represents the number of proteins and nd represents the number of diseases. Based on previous studies, we set the initial bandwidths $\rho_{p}^{\prime }$ and $\rho_{d}^{\prime }$ to 1.

In the context of this study, we define the disease similarity used in subsequent steps to construct the heterogeneous network as SD, which is equivalent to SGD, while the protein similarity is designated as SP. The integration techniques for these similarities are described as

(6) $$SP\left(p_{i},p_{j}\right)=\alpha_{1}SSP\left(p_{i},p_{j}\right)+\alpha_{2}SGP\left(p_{i},p_{j}\right)$$

where SSP denotes the sequence similarity matrix of proteins, and SGP represents the Gaussian interaction profile (GIP) kernel similarity matrix of proteins. In alignment with the methodology delineated in antecedent research, the parameters $\alpha_{i}$ for i∈{1,2} were assigned a value of 1 (22).

### Heterogeneous Network and Input Graph

We constructed a PTM-disease heterogeneous network by combining known PTM-disease associations, integrated protein similarity, and integrated disease similarity. The methodology for integrating these components into the heterogeneous network, denoted as $A_{H}$, is explained as:

(7) $$A_{H}=\left[\begin{array}{cc} D_{p}^{-\frac{1}{2}}SPD_{p}^{-\frac{1}{2}} & A\\ A^{T} & D_{d}^{-\frac{1}{2}}SDD_{d}^{-\frac{1}{2}} \end{array}\right]$$

where A represents the known PTM-disease associations matrix, and $A^{T}$ represents the transpose of matrix A. Here, $D_{p}$ and $D_{d}$ are diagonal matrices where $D_{p}=diag\left(\sum_{j}SP_{ij}\right)$ and $D_{d}=diag\left(\sum_{j}SD_{ij}\right)$, respectively. They can normalize the similarity for protein and disease within the heterogeneous network $A_{H}$, thereby harmonizing feature discrepancies and enhancing the model’s convergence rate.

To modulate the influence of similarity information and prevent overfitting, a penalty factor Ψ is incorporated into the heterogeneous network. The comprehensive input for the model, $G_{pd}$ is explicated in

(8) $$G_{pd}=\left[\begin{array}{cc} \Psi D_{p}^{-\frac{1}{2}}SPD_{p}^{-\frac{1}{2}} & A\\ A^{T} & \Psi D_{d}^{-\frac{1}{2}}SDD_{d}^{-\frac{1}{2}} \end{array}\right]$$

### PTMFusionNet

**Fig. 1:**
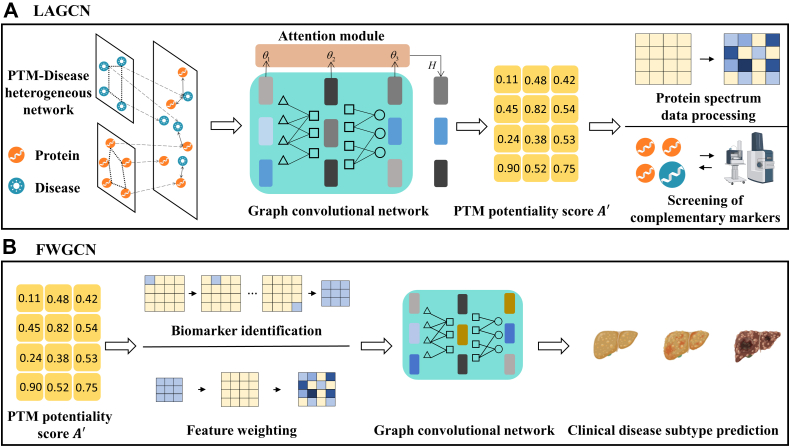
**The flowchart of PTMFuionNet**. *A*, LAGCN flowchart. We first integrate known PTM-Disease associations, protein similarity, and disease similarity to form a PTM-Disease heterogeneous network, which is then input into a graph convolutional network with layer-attention mechanisms to output PTM potentiality scores. Using these PTM potentiality scores, we subsequently process protein mass spectrometry data (specific processing methods shown in *B*) and screen supplementary biomarkers from mass spectrometry results. *B*, FWGCN flowchart. We use PTM potentiality scores to weight protein expression data, thereby filtering PTMs that improve results (defined as critical PTM biomarkers). Finally, we apply weighting to the entire protein expression data using potentiality scores of all critical PTM biomarkers. The weighted expression data is input into a GCN for training, ultimately yielding a model for precise disease subtype classification.

PTMFusionNet consists of two distinct Graph Convolutional Network (GCN) models, namely layer-attention graph convolutional network (LAGCN) and Feature Weighting Graph Convolutional Network (FWGCN). The procedural schematic of the PTMFusionNet model is depicted in Figure 1.

## LAGCN

The LAGCN is composed of an input layer, multiple hidden layers, and an output layer, with each layer adhering to the propagation rule delineated in

(9) $$H_{pd}^{\left(l+1\right)}=f\left(H_{pd}^{\left(l\right)},G_{pd}\right)=\sigma \left(D_{pd}^{-\frac{1}{2}}G_{pd}D_{pd}^{-\frac{1}{2}}H_{pd}^{\left(l\right)}W_{pd}^{\left(l\right)}\right)$$

where $H_{pd}^{\left(l\right)}$ denotes the node embedding of the $l_{th}$ layer, and $D_{pd}$ is the diagonal matrix corresponding to the input graph $G_{pd}$, defined as $D_{pd}=\text{diag}\left(\sum_{j}{\left(G_{pd}\right)}_{ij}\right)$. The trainable weight matrix for the $l_{th}$ layer is represented by $W_{pd}^{\left(l\right)}$, and σ(·) signifies a nonlinear activation function. The embedding for the initial layer is articulated in

(10) $$H_{pd}^{\left(0\right)}=\left[\begin{array}{cc} 0 & A_{H}\\ A_{H}^{T} & 0 \end{array}\right]$$

where $A_{H}$ represents the PTM-disease heterogeneous network. Within the LAGCN, each layer is capable of capturing distinct structural information. Given the variable significance of different layers to the embeddings, we employed a layer-attention mechanism to amplify the influence of the pivotal layers. The ultimate embeddings for protein p and disease $H_{d}$ are derived as

(11) $$\left[\begin{array}{c} H_{p}\\ H_{d} \end{array}\right]=\sum \theta_{l}H^{l}$$

where $\theta_{l}$ parameters were learned autonomously by the neural network, initialized as 1/(l+1),l=1,2,…,L. The PTM-disease association degree score matrix, denoted as $A^{\prime }$ is reconstructed utilizing a bilinear decoder as delineated in

(12) $$A^{\prime }=\text{sigmoid}\left(H_{p}W_{pd}^{\prime }H_{d}^{T}\right)$$

where $W_{pd}^{\prime }$ is a trainable matrix. The element $a_{i,j}$ within $A^{\prime }$ represents the PTM potentiality score between the $i_{th}$ protein and the $j_{th}$ disease.

### Optimization

In the construction of a predictive model for PTM-disease associations, the dataset comprising known PTM-disease associations was designated as the positive set $T^{+}$, while all unobserved PTM-disease pairs constituted the negative set $T^{-}$. The sample imbalance presented a challenge due to the disparity in size between the positive and negative sets. To rectify this, weighted cross-entropy was employed as the loss function, formalized as

(13) $$L_{pd}=-\;\frac{1}{P\times D}\left(\frac{\left|T^{-}\right|}{\left|T^{+}\right|}\;\times \sum_{\left(i,j\right)\in T^{+}}\log\left(a_{ij}^{\prime }\right)+\sum_{\left(i,j\right)\in T^{-}}\log\left(1-\;a_{ij}^{\prime }\right)\right)$$

where P and D denote the number of proteins and diseases, respectively, and (i,j) signifies the pair corresponding to the $i_{th}$ protein and the $j_{th}$ disease. We consider the protein-disease associations in dataset1 as positive instances, and other pairs as negative instances. Therefore, the cardinalities of the positive and negative sets are denoted as $\left|T^{+}\right|$ and $\left|T^{-}\right|$, respectively.

## FWGCN

In FWGCN, we first matched the PTM-disease known association dataset with the protein expression dataset to obtain a subset of shared proteins. Then, we weighted the protein expression data using the PTM potentiality score corresponding to each shared protein. We describe the weighting process in (Equation 14). Moreover, we improved the weighting process by using L1 regularization to reduce feature weights and mitigate the risk of overfitting. For a given expression datum $p_{i,j}$ between the $i_{th}$ protein and the $j_{th}$ disease, it was weighted according to $A_{i,j}$, which represents the association score between the $i_{th}$ protein and the $j_{th}$ disease, to yield the updated expression datum $p_{i,j}^{\prime }$, delineated as

(14) $$p_{i,j}^{\prime }=2*p_{i,j}*A_{i,j}*\exp\;\left(\delta *\left|I\left(p_{i,j}\right)\right|\right)$$

where a multiplicative factor of 2 was assigned for weighting. This factor indicates that the protein abundance metrics for a given disease will decrease when the potential association score drops below the threshold of 0.5. Conversely, an association score surpassing 0.5 prompts an increment in the protein abundance metrics (23). Additionally, we enhanced the process of feature weighting by applying L1 regularization, which employs a multiplicative strategy to curtail the magnitude of feature weights, thereby alleviating the propensity for model overfitting. The exponential function is represented by exp(·), while δ signifies the coefficient for regularization. The optimal value for δ was ascertained via a quintuple cross-validation methodology.

Following the initial layer weighting, we acquired the weighted protein expression data, which subsequently served as the input for FWGCN. Specifically, the propagation rule of PTMFusionNet is the same as that of LAGCN, so we only need to replace the original adjacency matrix $G_{pd}$ with $G_{pp}$ as shown in

(15) $$H_{pp}^{\left(l+1\right)}=f\left(H_{pp}^{\left(l\right)},G_{pp}\right)$$

where $G_{pp}$ is constructed by calculating the cosine similarity between pairs of nodes and edges with cosine similarity larger than a threshold ϵ are retained. Specifically, $G_{pp}\left[i,j\right]$ is the adjacency between the $i_{th}$ node and the $j_{th}$ node in the graph, can be calculated as

(16) $$G_{pp}\left[i,j\right]=\left\{ \begin{array}{cc} s\left(\frac{x_{i}\cdot x_{j}}{∥x_{i}{∥}_{2}∥x_{j}{∥}_{2}}\right), & \text{if}\;i\neq j\;\text{and}\;s\left(\frac{x_{i}\cdot x_{j}}{∥x_{i}{∥}_{2}∥x_{j}{∥}_{2}}\right)\geq \epsilon \\ 0, & \text{otherwise} \end{array}\right.$$

where $x_{i}$ and $x_{j}$ are the feature vectors of the $i_{th}$ node and the $j_{th}$ node in P, The threshold ϵ is determined given a parameter γ, which represents the average number of edges per node that are retained including self-connections:

(17) $$\gamma =\sum_{i,j}I\left(s\left(\frac{x_{i}\cdot x_{j}}{∥x_{i}{∥}_{2}∥x_{j}{∥}_{2}}\right)\geq \epsilon \right)/n$$

where I(·) is an indicator function and n is the number of nodes. For the adjacency matrix generation defined in (Equation 17), the hyperparameter γ was systematically optimized across candidate values {2,5,10} through empirical evaluation on training data, with the selected γ value remaining consistent for all experimental trials within each dataset. In this study, we further extend the application of PTMFusionNet to supervised classification tasks. For the training data $X_{tr}$, the corresponding adjacency matrix $G_{pp}^{tr}$ can be computed from (Equation 16). Then, a graph convolutional network can be used to train on $X_{tr}$ and $G_{pp}^{tr}$, and the predictions for the training data can be expressed as

(18) $${\hat{Y}}_{tr}=\text{GCN}\left(X_{tr},G_{pp}^{tr}\right)$$

where the ${\hat{Y}}_{tr}$ represents the predicted label probability for the training sample. For a new test sample $X_{te}$, we add it to the training set, extending $X_{tr}$ to $X_{tr,te}$, and generate the extended adjacency matrix $G_{pp}^{tr,te}$ according to (Equation 16). Therefore, given $X_{tr,te}$, $G_{pp}^{tr,te}$ and the trained model GCN(·), we can obtain ${\hat{Y}}_{tr,te}=GCN\left(X_{tr,te},G_{pp}^{tr,te}\right)$. The predicted label probability distribution for the test sample is the last row of ${\hat{Y}}_{tr,te}$. Hence, by exploiting both the features of the test sample and the association between the test sample and the training samples, we can predict the label of the new test sample $X_{te}$. Overall, the loss function of PTMFusionNet can be written as

(19) $$L_{pp}=\sum_{j=1}^{n_{tr}}L_{CE}\left({\hat{y}}_{j},y_{j}\right)=\sum_{j=1}^{n_{tr}}-\log\left(\frac{e^{{\hat{y}}_{j}\cdot y_{j}}}{\sum_{k}e^{{\hat{y}}_{j,q}}}\right)$$

where $L_{CE}\left(\cdot \right)$ denotes the cross-entropy loss function. $y_{j}$ is the true label of the $j_{th}$ training sample, and ${\hat{y}}_{j,q}$ is the $q_{th}$ element of the vector ${\hat{y}}_{j}$.

### Hyperparameter Configuration

The hyperparameters of PTMFusionNet were optimized through grid search and empirical validation. For the LAGCN model, we finally determined the embedding dimension k=64, the convolutional layer number L=3, the initial learning rate lr=0.001, the total training steps α=400, the two dropout ratios ϕ=0.6 and ω=0.4, and the penalty factor Ψ=6. In the FWGCN, the value of δ was determined within a specified range of δ∈{0.0001,0.0003,0.001,0.003,0.01,0.03,0.1,0.3,1,3} through the 5-fold cross-validation.

## Results

### Evaluation of Performance in Predicting Potential PTM

To assess the degree of PTM-disease association, we utilized a feature learning paradigm based on LAGCN. Equipped with a layer-attention mechanism, LAGCN adeptly captures multi-level information within the graph structure, effectively addressing the complexity of heterogeneous networks. Due to the limited number of PTM-disease associations in dataset2, we performed 5-fold cross-validation on dataset1 to evaluate LAGCN’s performance in predicting potential association scores and to determine its key parameters.

**Fig. 2:**
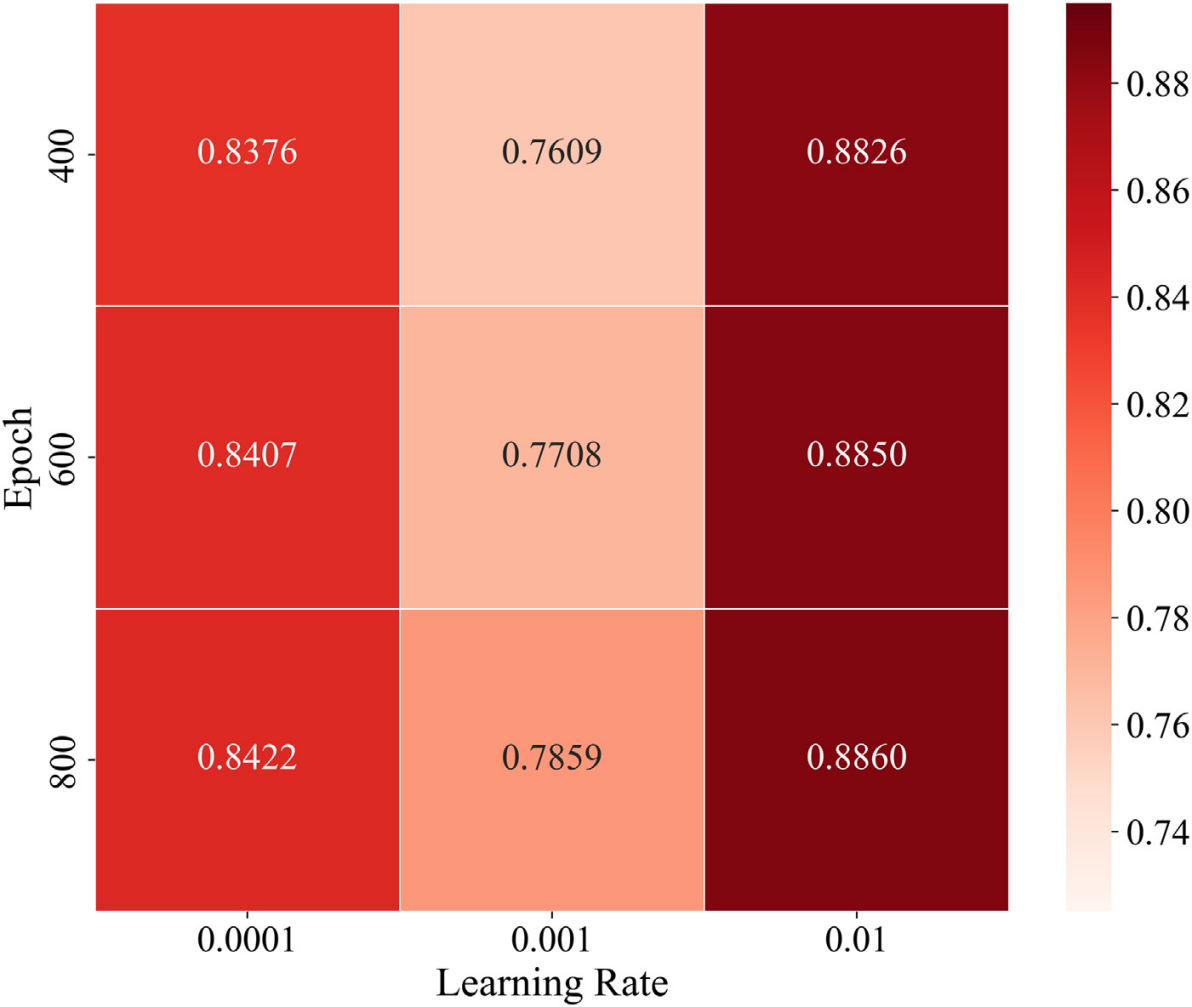
**AUC values for different training steps and learning rates.** Heatmap showing AUC values from grid searches with different epochs and learning rates when using LAGCN to predict PTM potentiality scores. LAGCN achieves the highest AUC value of 0.8860 (optimal prediction performance) at Epoch = 800 and Learning Rate = 0.01.

Specifically, we randomly divided all known PTM-disease associations into five approximately equal parts. In each iteration, one part served as the test sample, while the remaining four parts were used as training samples, with all unknown PTM-disease pairs as candidate samples. After partitioning, LAGCN scored the test and candidate samples. After dividing the original known associations into training and test sets, we masked the test set entries in the adjacency matrix A representing the dataset by setting their original "1" elements to "0". Based on these scores, we ranked the test samples among all candidate samples based on these scores. If a test sample’s ranking exceeded a set threshold, LAGCN was considered to have made an accurate prediction. Subsequently, Receiver Operating Characteristic (ROC) curves were plotted under different thresholds based on the rankings of all test samples, and the Area Under the ROC Curve (AUC) was calculated to assess the performance of LAGCN. Previous studies have established some basic parameters for this model, and the dataset used is substantial. Therefore, we only considered two key parameters: total training steps α∈{400,600,800} and learning rate lr∈{0.0001,0.001,0.01}. By implementing grid search within this range, we performed 5-fold cross-validation for each round and recorded the averages. Finally, we plotted the results as a heatmap, as shown in Figure 2. By comparing AUC values, we determined the optimal values to be lr=0.01 and α=800.

### Evaluation of Performance in Disease Classification

After deriving the PTM potentiality score through initial fusion, we weighted the protein expression dataset to enhance cancer classification methods. We compared the classification performance of PTMFusionNet with the following three existing omics data classification algorithms (1): K-nearest neighbor classifier (KNN): Label predictions were made by voting of KNN in the training data (2). Support vector machine classifier (SVM): The preprocessed data was used as input for the SVM to predict the sample labels in the test set (3). IBPGNET (24): Developed by Xu *et al*., IBPGNET is an interpretable network for disease recurrence prediction and related biomarker discovery. IBPGNET has been proven to outperform nine benchmark methods across various omics data. Therefore, we selected it as a comparative algorithm on protein expression data to demonstrate the superior performance of PTMFusionNet.

Specifically, we used three measures to assess PTMFusionNet’s effectiveness in cancer subtype classification: for cancers with two subtypes, we used accuracy (ACC), F1 score (F1), and AUC; for cancers with three subtypes, we used ACC, the weighted average F1 score (F1_weighted), and the macro-average F1 score (F1_macro).

To validate PTMFusionNet’s effectiveness, we used 5-fold cross-validation. The dataset was randomly divided into five roughly equal subsets, with each subset serving as the test set once, while the remaining four subsets were used as the training set. We applied this method to all approaches and reported the average and standard deviation of the 5-fold cross-validation. Our comparative analysis across multiple datasets demonstrated that PTMFusionNet outperformed several benchmark algorithms in all evaluations. This underscores the effectiveness of the identified key protein biomarkers and establishes PTMFusionNet’s superiority in disease subtype classification. Table 2, Table 3, Table 4 present the comparative results for the COADREAD, KIPAN, and THCA datasets, respectively.

**Table 2:** Classification results on COADREAD dataset

Method	ACC	F1	AUC
SVM	0.6046 ± 0.0505	0.6136 ± 0.0633	0.5744 ± 0.0546
KNN	0.6595 ± 0.0324	0.6147 ± 0.0545	0.6189 ± 0.0381
IBPGNET	0.7002 ± 0.0175	0.6717 ± 0.0547	0.6856 ± 0.0457
PTMFusionNet	0.8227 ± 0.0528	0.8157 ± 0.0555	0.7558 ± 0.0671

**Table 3:** Classification results on KIPAN dataset

Method	ACC	F1_weighted	F1_macro
SVM	0.8308 ± 0.0236	0.8274 ± 0.0562	0.8217 ± 0.0106
KNN	0.8508 ± 0.0100	0.8500 ± 0.0096	0.8272 ± 0.0103
IBPGNET	0.9493 ± 0.0073	0.9483 ± 0.0065	0.9223 ± 0.0104
PTMFusionNet	0.9666 ± 0.0029	0.9657 ± 0.0030	0.9364 ± 0.0225

**Table 4:** Classification results on THCA dataset

Method	ACC	F1	AUC
SVM	0.6732 ± 0.0342	0.4023 ± 0.0683	0.5204 ± 0.0646
KNN	0.7071 ± 0.0368	0.4363 ± 0.0854	0.5771 ± 0.0659
IBPGNET	0.6875 ± 0.0590	0.4431 ± 0.0286	0.6709 ± 0.0350
PTMFusionNet	0.7440 ± 0.0622	0.4909 ± 0.1247	0.6885 ± 0.0994

### Ablation Experiments with Feature Weighting Module

**Fig. 3:**
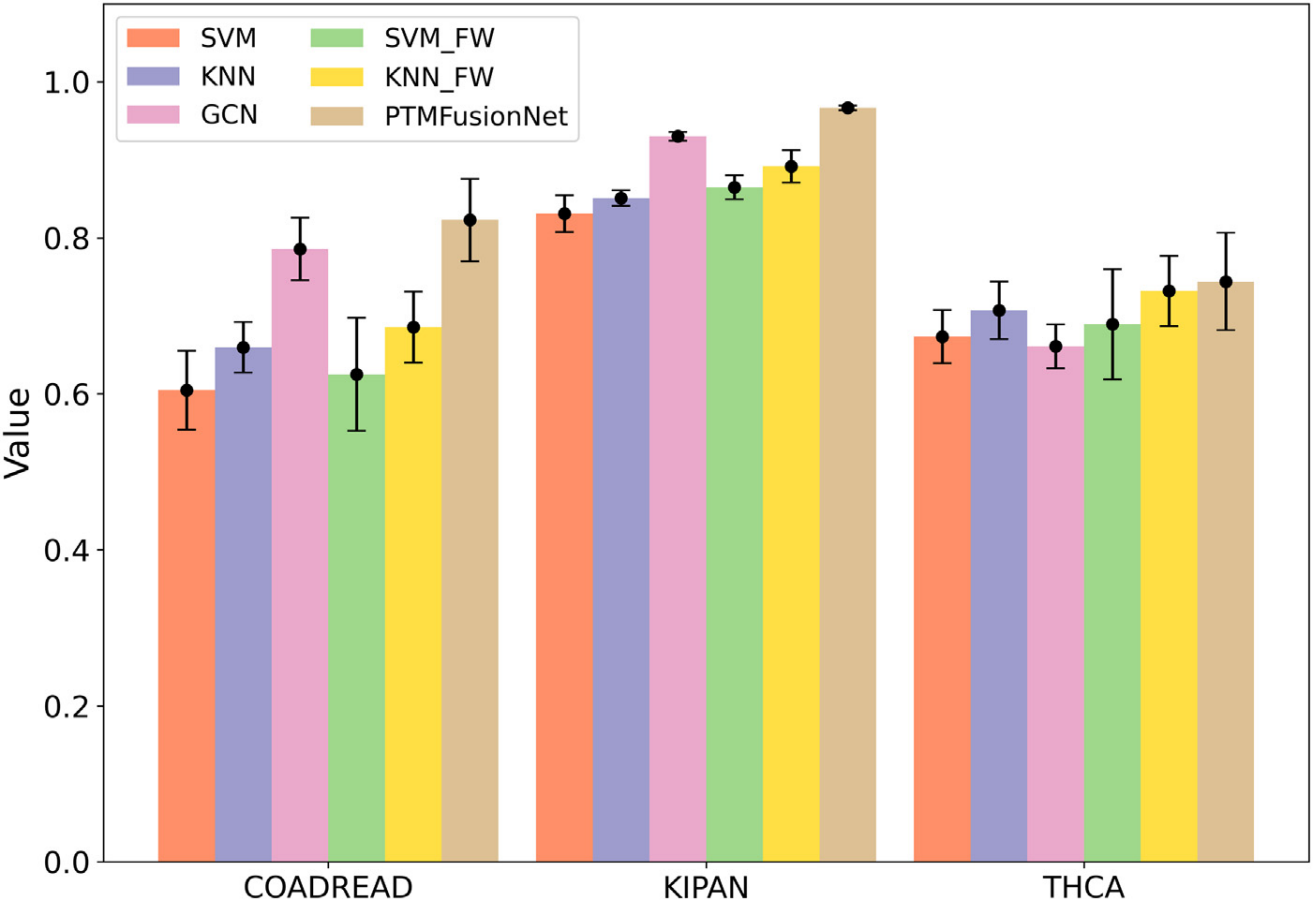
**Impact of Feature Weighting on Classification Accuracy.** Bar chart showing ACC results of different models with/without FW (Feature Weighting) processing. All FW-enhanced models outperform their baseline counterparts. SVM_FW denotes SVM with FW processing, and KNN_FW represents KNN with FW processing. PTMFusionNet degenerates into a regular GCN when the FW processing layer is removed.

In PTMFusionNet, feature weighting is the most critical strategy, integrating LAGCN and FWGCN. To assess its effectiveness, we performed comprehensive ablation experiments on three datasets: COADREAD, KIPAN, and THCA. Specifically, we excluded the feature weighting process from FWGCN, using only GCN to evaluate the datasets and calculate the classification ACC for these cancers. The findings revealed that FWGCN with feature weighting significantly enhanced classification accuracy compared to GCN without it. Furthermore, we incorporated the feature weighting strategy into several traditional machine learning algorithms. The results demonstrated that the classification performance of these algorithms improved markedly with the feature weighting strategy, confirming its effectiveness. We documented the ACC improvements for each algorithm across the three datasets before and after applying the feature weighting strategy, as illustrated in Figure 3.

### Impact of Shared Protein Quantity on PTMFusionNet Performance

The overlap between PTM-disease associations and protein expression datasets is limited. In disease datasets with fewer protein types, it is highly likely that only a small number or even no shared proteins exist. Under such extreme circumstances, the performance of the current version of PTMFusionNet may be compromised.

**Fig. 4:**
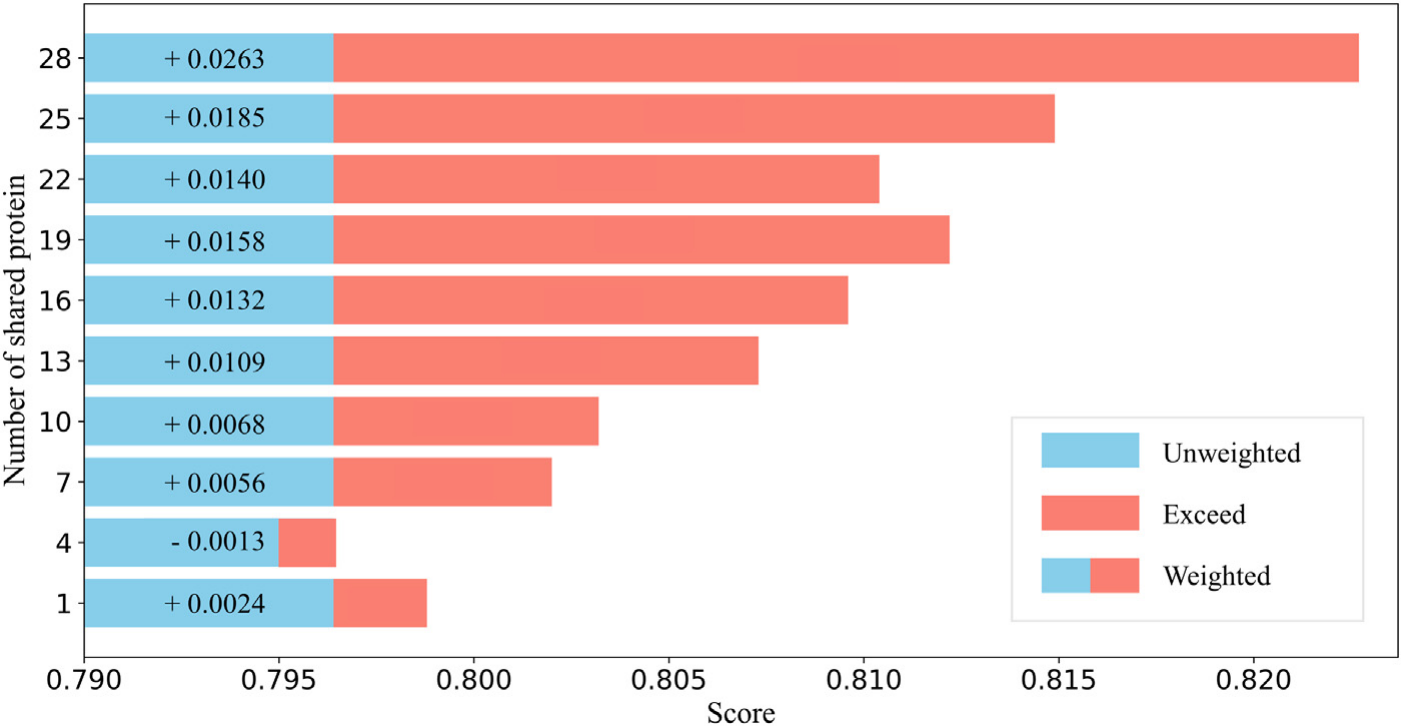
**Impact of Different Numbers of Shared Proteins on Disease Subtype Prediction Results.** Bar chart showing the impact of shared protein type quantities on PTMFusionNet's ACC for disease subtype classification. *Blue* bars show ACC without FW strategy (shared protein types=0). *Red* bars indicate ACC improvements exceeding baseline when effective FW is applied.

To investigate the relationship between the number of shared proteins and disease subtype classification predictions, we randomly reduced the number of shared proteins in the COADREAD dataset and plotted a line chart of ACC variation in disease subtype prediction results. As shown in Figure 4, the overall predictive performance of PTMFusionNet exhibited an increasing trend as the number of shared proteins grew. However, when the number of shared proteins was extremely low, PTMFusionNet’s performance might underperform compared to GCN. For instance, in the case where the y-axis value in Figure 4 is 4, the weighted prediction results were lower than the unweighted results. Overall, the limited yet inevitable overlap between PTM-disease associations and protein expression datasets implies that we recommend using PTMFusionNet when the number of shared proteins exceeds 10, as this minimizes prediction degradation caused by stochastic errors.

### Validation of Protein Similarity Fusion and Layer-Attention Mechanism

**Fig. 5:**
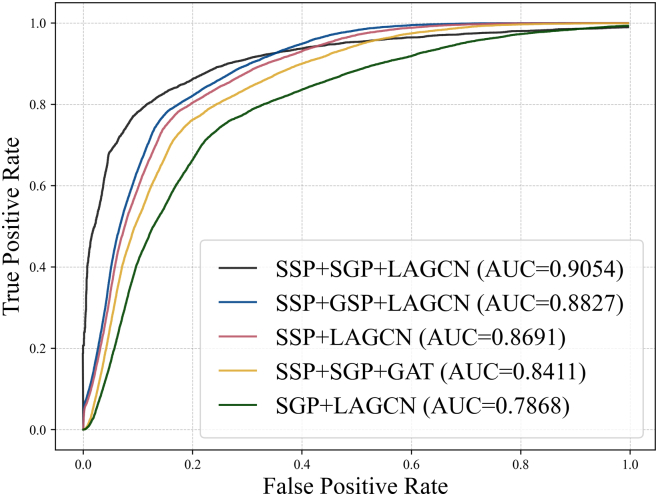
**Performance evaluation of PTMFusionNet with varying similarity metrics and attention mechanisms.** Line chart showing ROC curves of PTMFusionNet versions with different component replacements. The current version (SSP + SGP + LAGCN) achieves optimal performance.

In constructing the PTM-disease heterogeneous network, we integrated sequence similarity of protein (SSP) and Gaussian Interaction Profile kernel similarity (GIP) to characterize sequence conservation and functional associations. Ablation experiments demonstrated that the SSP and GIP fusion strategy achieved an AUC of 0.9054, significantly outperforming the use of SSP alone with an AUC of 0.8691 or GIP alone with an AUC of 0.7868, confirming their complementary roles. Gene Ontology-based functional similarity (GSP) exhibited an inferior AUC of 0.8827 compared to GIP due to incomplete annotation coverage and limited overlap with the dataset, as shown in Figure 5.

Regarding model architecture, the Layer Attention Graph Convolutional Network, LAGCN, achieved an AUC of 0.9054, outperforming the Graph Attention Network (GAT), which yielded an AUC of 0.8411. While GAT overly focuses on local node relationships, such as known PTM-disease associations and risks local optimization, LAGCN dynamically balances shallow topological features, like sequence conservation and deep semantic patterns through its layer-attention mechanism, enabling global multi-scale modeling. Attention weight analysis further revealed that shallow features contributed dominantly to PTM prediction, aligning with the biological observation that PTM sites often reside in evolutionarily conserved regions.

In summary, the fusion of SSP and GIP combined with the layer-attention mechanism in LAGCN synergistically enhanced the predictive performance and interpretability of PTMFusionNet by integrating multidimensional features and enabling dynamic modeling. This framework provides an efficient solution for disease subtype classification and biomarker discovery.

### Important PTM Biomarker Related to COADREAD

To identify significant PTM biomarkers in the COADREAD, we matched the shared proteins between protein expression data and PTM-disease association data. Next, we implemented a 5-fold cross-validation method on all dataset samples. The dataset was divided into five equally sized subsets, with one subset used as the validation set and the remaining four subsets used as the training set in each iteration. We assigned feature weights to the expression levels of each protein based on their PTM potentiality score. If the weighting of a PTM potentiality score led to an improvement in predictive performance, it was considered an important PTM biomarker and recorded. This process was repeated five times, and then we aggregated the results, categorizing the selected biomarkers based on their frequency of occurrence. PTM biomarkers with higher occurrence frequencies across cross-validation folds were prioritized as more significant.

In the biomarker screening of the COADREAD dataset, 5-fold cross-validation identified 52 significant PTM biomarkers. After removing duplicates, 26 unique PTMs remained. The frequency of these PTMs ranged from one to five occurrences, with 7, 14, 3, 2, and 0, respectively. The most notable potential PTMs appeared four times, associated with the proteins PREX1 and SMAD3.

The PTMs of the PREX1 protein include phosphorylation, acetylation, and ubiquitination, which regulate its stability, localization, and interactions with other proteins. In colorectal cancer, the expression level of PREX1 is significantly elevated, and its PTMs are closely related to the migration and invasion capabilities of cancer cells. Specifically, the phosphorylation state of PREX1 was mapped to Rho GTPase regulation, a pathway experimentally proven to drive cytoskeletal remodeling and metastatic invasion in colorectal cancer (25, 26). Additionally, high expression of PREX1 is correlated with poor prognosis, indicating its important role in the progression of colorectal cancer (27).

The PTMs of the SMAD3 protein include phosphorylation, acetylation, methylation, and ubiquitination, which regulate its activity, stability, and interactions with other proteins. SMAD3 plays a key role in the TGF-β signaling pathway, and its PTMs are significant in the progression of colorectal cancer (28, 29). Studies have shown that the phosphorylation state of SMAD3 is associated with increased proliferation, migration, and invasion of cancer cells, and its acetylation and methylation modifications are also related to increased invasiveness and metastasis of cancer cells (30, 31). High expression of SMAD3 is correlated with poor prognosis, indicating its important role in the progression of colorectal cancer (31, 32).

The translational relevance of PTMFusionNet is significantly strengthened by explicitly bridging learned PTM features with mechanistically validated pathways. For instance, the model-predicted phosphorylation of PREX1—a high-potential biomarker—was mapped to Rho GTPase regulation. Similarly, SMAD3 phosphorylation, prioritized by PTMFusionNet, was anchored to TGF-β signaling. By directly linking these data-driven PTM patterns to precisely characterized biological mechanisms, we not only validated the model’s clinical reliability but also identifies actionable targets for therapeutic intervention, demonstrating how AI-driven biomarker discovery can accelerate translational oncology.

### Case Study by Literature and Mass Spectrometry Data

To validate the effectiveness of PTMFusionNet in identifying critical protein biomarkers and predicting potential PTM-disease associations, we analyzed published phosphoproteomic data from extracellular vesicles (EVs) in cerebrospinal fluid (CSF) samples of primary central nervous system lymphoma (PCNSL) patients and urine samples of prostate cancer patients. In the referenced studies, Deng *et al.* isolated EVs from CSF samples through centrifugation and functionalized magnetic bead capture (EVTRAP), followed by mass spectrometry-based phosphoprotein quantification for PCNSL detection (33). Similarly, Sun *et al.* employed bifunctional magnetic beads (BiMBs) for EV isolation of urine from prostate cancer patients, subsequently performing nano-liquid chromatography-mass spectrometry phosphoproteomic analysis (34). These well-established experimental methodologies provide reliable datasets for investigating neurological and prostatic disorders.

In the known PTM-disease association dataset, no PTM-disease associations are identified for proteins related to PCNSL. Proteins were ranked according to their PTM potentiality score, and the top 20 proteins were selected. In addition to LC-MS/MS validation, we also conducted literature validation for proteins with potential PTMs. Eight proteins were confirmed to have PTMs, as detailed in Table 5.

**Table 5:** LC-MS/MS and literature validation of proteins with potential PTMs in PCNSL

Rank	Protein	Evidence	Rank	Protein	Evidence
1	STAT3	LC-MS/MS	11	HSPA1A	unconfirmed
2	CDKN1B	PMID: 33316141	12	EGFR	PMID: 31485130
3	HIST1H3A	unconfirmed	13	CTNNB1	unconfirmed
4	TP53	unconfirmed	14	EIF4EBP1	unconfirmed
5	AKT1	PMID: 38860522	15	STMN1	LC-MS/MS
6	GSK3B	LC-MS/MS	16	PXN	PMID: 34135128
7	MYC	unconfirmed	17	EIF2S1	unconfirmed
8	PTK2	unconfirmed	18	STAT5A	PMID: 32872372
9	SRC	unconfirmed	19	MAPT	unconfirmed
10	RB1	unconfirmed	20	HIST1H4A	unconfirmed

In the known PTM-disease association dataset, there are 43 known PTM-disease associations related to prostate cancer proteins. We excluded these 43 known PTM-disease associations and only considered PTM-disease pairs without known associations. There are 19 proteins in the top 20 that have been confirmed to have PTMs through literature validation. Details are shown in Table 6.

**Table 6:** LC-MS/MS and literature validation of proteins with potential PTMs in prostate cancer

Rank	Protein	Evidence	Rank	Protein	Evidence
1	EPHA2	PMID: 30797819	11	STAT5A	PMID: 26026053
2	JUN	PMID: 37271807	12	YAP1	PMID: 36979713
3	MAPT	PMID: 37000265	13	EIF2S1	PMID: 29329780
4	RYR2	PMID: 36720353	14	GSK3B	PMID: 26934497
5	MET	PMID: 25862631	15	DNM1L	LC-MS/MS
6	STMN1	PMID: 26986925	16	E2F1	PMID: 31870703
7	STAT5B	PMID: 38341429	17	PRKAA1	PMID: 27103440
8	HSPA1A	PMID: 27511022	18	TNNI3	unconfirmed
9	PXN	PMID: 32139877	19	EZH2	PMID: 34093859
10	EIF4EBP1	PMID: 29453322	20	RPS6KB1	PMID: 38240100

### Performance of PTMFusionNet with Hyperparameters δ

**Fig. 6:**
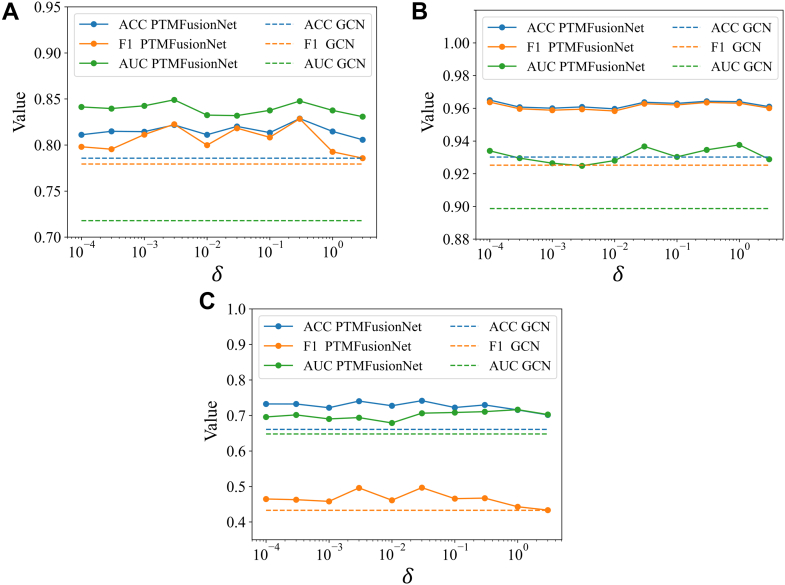
**Performance of PTMFusionNet under different values of hyperparameter**δ. *A*, COADREAD dataset results. *B*, KIPAN dataset results. *C*, THCA dataset results. *Dashed lines* represent GCN results (classification model without FW method). PTMFusionNet outperforms GCN across different δ values.

In the intermediate fusion phase of the PTMFusionNet framework, which leverages the association degree score matrix, the regularization coefficient plays a crucial role. Applying regularization to the weighted protein expression data enhances the model’s effectiveness and clarity. This coefficient is key in controlling the sparsity of essential biological markers, influencing the model’s adaptability to new datasets. A low regularization coefficient might retain unnecessary markers, increasing the model’s complexity. Conversely, a high coefficient could overlook important markers with significant association scores, reducing the model’s efficacy. Therefore, precise calibration of the regularization coefficient is vital for optimizing PTMFusionNet’s performance. The ideal regularization coefficient depends on the intrinsic properties of the data and the PTM-disease association degree scores, which vary across different datasets. In our study, we determined the regularization coefficient through 5-fold cross-validation on the training set. To assess the impact of the regularization coefficient on PTMFusionNet’s performance in binary and multiclass classifications, we conducted multiple trials on the COADREAD, KIPAN, and THCA datasets. As shown in Figure 6, the regularization coefficient influences PTMFusionNet’s classification ability, with results fluctuating as the coefficient is adjusted. Additionally, across these datasets, the ACC and F1_weighted metrics (with F1 relevant to COADREAD) were generally consistent, indicating balanced performance by PTMFusionNet without significant bias.

## Discussion

With the advent of high-throughput proteomics detection methods such as mass spectrometry, it has become possible to simultaneously measure protein expression levels and post-translational modification (PTM) information on a large scale. This advancement enables the application of machine learning in the joint analysis of protein expression data and PTMs. In this study, we introduce a novel method called PTMFusionNet, which infers a PTM potential score from known protein-disease associations and their PTM information. This score represents the likelihood of PTMs existing between a given protein-disease pair. Using the PTM potential score, we employ a feature weighting strategy to weight patients’ protein expression data, thereby improving the accuracy of disease subtype diagnosis and identifying important PTM biomarkers.

The design of PTMFusionNet cleverly utilizes a feature weighting strategy to integrate PTM information with protein expression data, leveraging PTM prior knowledge to guide proteomics. This integration enhances the application of proteomics in clinical diagnosis and treatment, expanding the scenarios for PTM application. Moreover, layer-attention technology is applied in the LAGCN model, further screening important protein features, making the model more focused on proteins that play crucial roles in disease progression, and enhancing the interpretability of PTMFusionNet.

Despite the progress PTMFusionNet has made in the joint analysis of PTM and protein expression data, it still faces ongoing challenges and limitations. Firstly, the scarcity of data, with a low proportion of proteins present in both PTM-disease association datasets and protein expression datasets, limits the range of features that can be weighted. Secondly, PTMFusionNet currently can only predict the presence or absence of PTMs, not the specific types and sites of PTMs, which limits its depth of application in precision medicine. This simplification stems from the inherent annotation bias in current PTM-disease databases and the sparse coverage of non-canonical PTM types. Nevertheless, the prioritized PTM biomarkers provide high-confidence candidates for experimental validation, significantly reducing the cost of large-scale screening. Future work is likely to focus on constructing comprehensive datasets of protein and other omics features, rich in PTM types and site information, to form a multi-omics prior knowledge base applicable to clinical research projects.

## Data Availability

The mass spectrometry proteomics data (including raw instrument files) are publicly accessible via the ProteomeXchange Consortium: CSF dataset (PXD040744/JPST002076) through JPOST, and prostate cancer dataset (PXD020573) through PRIDE. Omics data and codes were obtained from The Cancer Genome Atlas Program (TCGA). The known PTM-disease association was are available at (https://github.com/Jie-Ni/PTMFusionNet).

## Conflict of interest

The authors declare that they have no conflicts of interest with the contents of this article.
